# Comparison of stiffness, elasticity and resilience values of ACL with autografts used instead of ACL in terms of texture analysis

**DOI:** 10.1016/j.heliyon.2024.e25588

**Published:** 2024-02-04

**Authors:** Ahmet Mert, Selim Çınaroğlu, Murat Aydın, Fatih Çiçek, Faruk Gazi Ceranoğlu

**Affiliations:** a Department of Orthopedics and Traumatology, Faculty of Medicine, Niğde Ömer Halisdemir University, TR-51240, Niğde, Turkey; b Department of Anatomy, Faculty of Medicine, Niğde Ömer Halisdemir University, TR-51240, Niğde, Turkey

**Keywords:** Anterior cruciate ligament, Autograft, Texture profile analysis, Knee, Reconstruction

## Abstract

**Background:**

Anterior cruciate ligament (ACL) ruptures are one of the most extensively studied injuries in the field of orthopedics, but despite the extensive research, graft selection for ACL reconstruction remains a matter of debate. The present study aims to evaluate the stiffness and elasticity of native ACLs using texture profile analysis and compare results with those of autografts used in ACL reconstruction.

**Methods:**

Included in the study were dissected 12 cadavers from which grafts were recovered. The graft characteristics, such as stiffness, elasticity and resilience, were measured using a TA.XT Plus Texture Analyzer.

**Findings:**

Among the grafts studied, Achilles’ tendon graft (0.70; 0.64) had the highest resilience in both males and females, while the greatest elasticity was identified in the patellar tendon graft (Male: 93 %; Female: 94 %) in all subjects. The highest stiffness value in males was recorded for the quadriceps tendon graft (2928.76 N), while the highest stiffness value in females was recorded for Achilles’ tendon graft (2204.61 N).

**Interpretation:**

According to the study data, the autografts that may be considered as an alternative to ACL were, listed in order of strength from high to low, the quadriceps, Achilles’, patella and hamstring tendons in men, and the Achilles’, quadriceps, patellar and hamstring tendons in women. It is worthy of note that the hamstring tendon graft, which is the most frequently preferred autograft in ACL reconstruction, was found to be the lowest in all parameters in both groups.

## 1 Introduction

Knee injuries account for approximately 60 % of all sport-related injuries, and more than 50 % of these injuries occur in the cruciate ligaments [1]. Anterior cruciate ligament (ACL) tears occur most frequently in young and active people and can have long-term negative physical effects [2].

The ACL, one of the main ligaments in the human knee, has the primary function of limiting the anterior movement of the knee and providing joint stability by preventing the lateral dislocation of the femur from the tibia [2,3].

Surgical intervention (ACL reconstruction) is the preferred treatment in cases of anterior cruciate ligament tear, since nonsurgical interventions frequently result in failure [4]. Allografts, autografts and synthetic grafts have been used in the reconstruction of the anterior cruciate ligament [5], although autografts would seem to be superior to allografts in ACL reconstructions in active patients [2,6]. The Patellar and hamstring tendons (semitendinosus and gracilis tendons) are the most commonly preferred grafts for ACL reconstruction [4], although the Achilles’, quadriceps, patellar and hamstring tendons (semitendinosus and gracilis tendons), tibialis anterior tendons, tibialis posterior tendons, and peroneus longus tendons have been used as grafts in anterior cruciate ligament reconstruction procedures [5,7].

A sample’s texture is generally defined as its mechanical, geometrical and surface properties, and can be perceived through tactile, visual and auditory receptors. Texture analysis devices measure the sensorial properties of tissues and food mechanically [8,9]. Touch (tactile sense) is the first sense through which texture is measured, and tests involving cutting-penetration, tension-relaxation, extrusion, bending, stretching-twisting and texture profile analyses (TPA) aid in the optimization of tests aimed at determining, for example, the sensorial properties of a sample. Texture analysis devices (TA.XT Plus Texture Analyser Device) measure the stiffness, cohesiveness, elasticity, fracturability, gumminess, chewiness and resilience of a sample by touching, penetrating and applying pressure to the tissue [8,10,11]. The data were obtained mainly through sensorial analyses. Muscles that are harder to cut or penetrate assigned higher values.

Stretching and bending tests examine the deformation capacity of a material and are used in engineering to gain basic design information about materials related to their endurance [12].

The present study compares the autografts used in ACL reconstruction with native ACL based on a texture profile analysis. For this purpose, for the first time in the literature, the stiffness, elasticity and resilience values of the grafts (8 mm diameter) used for ACL reconstruction and the ACL were tested using tissue analyzers.

## 2 Methods

### 2.1 Ethical approval

The study was granted approval by the Ethics Committee of Niğde Ömer Halisdemir University (Protocol number: 2021/95).

### 2.2 Data sources

**Fig. 1:**
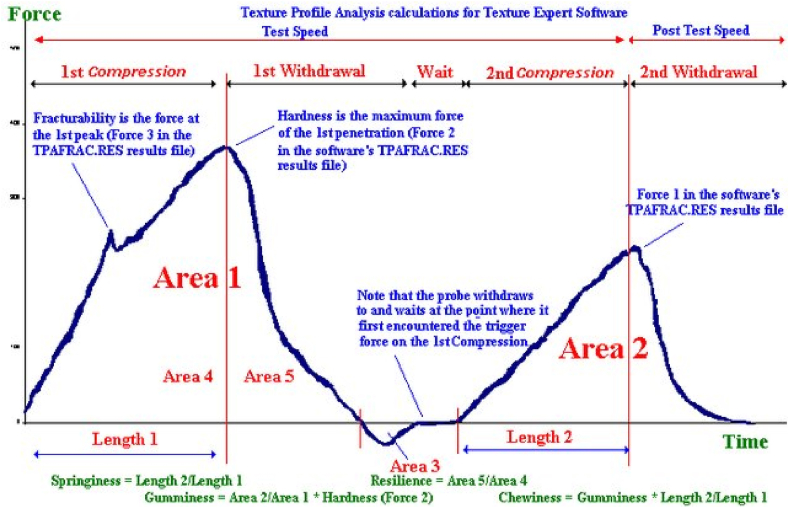
TPA graphic and interpretation [14].

**Texture profile analyses (TPA):** TPA are generally carried out to assess the structural, mechanical, and surface properties of food products, for which forces are applied to the sample to be analyzed, and the reactions of the sample, such as force and deformation time, are recorded and digitalized using a texture analyzer (Fig. 1) [13].

The main texture parameters, including stiffness, cohesiveness, elasticity, fracturability, gumminess, chewiness and resilience, are calculated from the curve produced by the TPA [[9], [10], [11],^15^]. The testing conditions, sample size and the direction of placement of the sample on the device are of utmost importance in a TPA analysis. A sample placed horizontally on the table of the instrument comes into contact with a probe moving downward from the instrument. When the trigger force or sensitivity 5 g pressure occurs, the measurement starts and is performed according to the established test procedure. If the size of the sample is smaller than 2 mm, the test cannot be performed because the probe of the device cannot apply pressure on the sample in the vertical axis and cannot penetrate. Sample length is not important in TPA analysis [16]. The textural features evaluated depending on the thickness/size of the ACL or the grafts used in place of the ACL are as follows:

**Stiffness:** “Stiffness”, obtained from the first compression curve, is defined as the force required to deform the sample by a certain degree, or the maximum force required for the first compression [[9], [10], [11]]. The stiffness value of the ACL and the grafts used in place of the ACL was the parameter selected to be determined digitally first in the present study. Stiffness is generally expressed in Newtons (N), although it can also be expressed in grams or kilograms [12].

**Elasticity:** In texture properties, it is the disappearance of the deformation that occurs after any effect on a sample when the effect is removed. In other words, it is the rate at which the texture returns to its former state after a force is applied to a sample [[8], [9], [10], [11]].

In the TPA graph, it is shown on the time line as the ratio of the 2nd Compression (Length 2: L2) to the first compression (Length 1: L1) (2nd Compression time/1st Compression time or L2/L1) (Fig. 1) [14]. Elasticity refers to the capacity of the sample tissue to reconstruct after compression, and so is a marker of the return of the sample to its original status. It has no units, being expressed as a percentage (%).

**Resilience:** Resilience refers to the ability of a tissue/material to respond to applied force [9,10,13]. In this study, resilience was calculated as the ratio of the area formed after the maximum peak force during the first compression cycle (Area5: A5) to the positive force area under the first compression (Area 4: A4), using the formula of A5/A4 (Fig. 1) [17]. It is a measure of how well a sample can recover from deformation at different forces and speeds. This parameter can only be measured at low test speeds, and it does not have a specific unit of measurement.

**Fig. 2:**
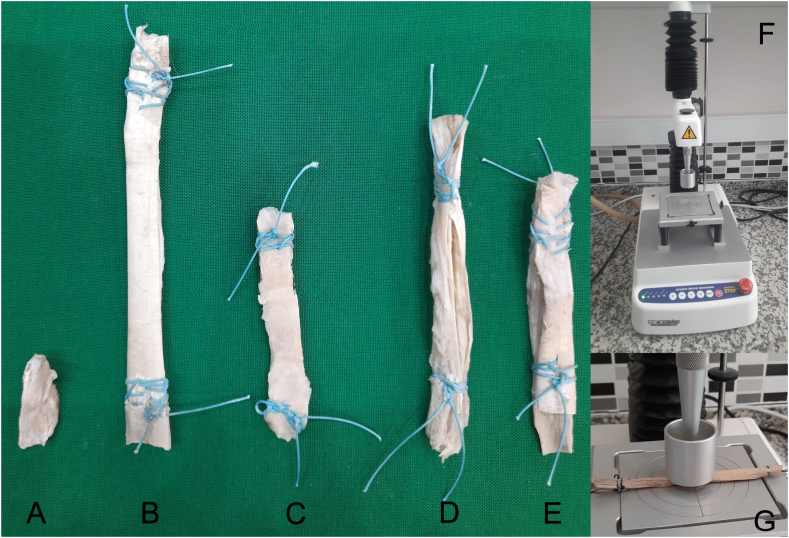
**A:** ACL (Anterior cruciate ligament), **B:** Achilles tendon, **C:** Patellar tendon, **D:** Hamstring tendon (Semitendinosus and gracilis tendons), **E:** Quadriceps tendon, **F:** TA.XT Plus Texture Analyser Device, **G:** Graft sample for test.

These parameters, as examined by TPA, can be summarized biomechanically as follows:

The stiffness value alone does not convey a meaningful interpretation, but rather represents the tissue's hardness value. Biomechanically, stiffer and more elastic tissues tend to be more resilient, depending on their function and role in the body. The goal of this study was to identify the graft with the highest desired values of stiffness, elasticity, and resilience modulus during sudden movements, which could be used as an alternative to the ACL. The texture analysis was performed using a TA.XT Plus Texture Analyzer Device (TA.XT Plus Texture Analyser Device. Stable Microsystems LTD, UK) in the Faculty of Engineering of Niğde Ömer Halisdemir University (Fig. 2).

**Test procedures:** A compression disc probe was used for the texture analysis (Fig. 2F), during which some basal parameters were accepted as follows: Pretest speed 2 mm/s, Post speed: 5 mm/s, Test speed: 2 mm/s, Target mode: 2 mm (Distance: Distance to enter the tissue vertically), Trigger force: 5 g and Load cell: 5 kg. The samples were then placed at the target point of the measuring table and secured with Ethibond sutures (Fig. 2G). To minimize the impact of errors, each sample was measured three times at 10-min intervals, and the average of the measurements was recorded.

**Fig. 3:**
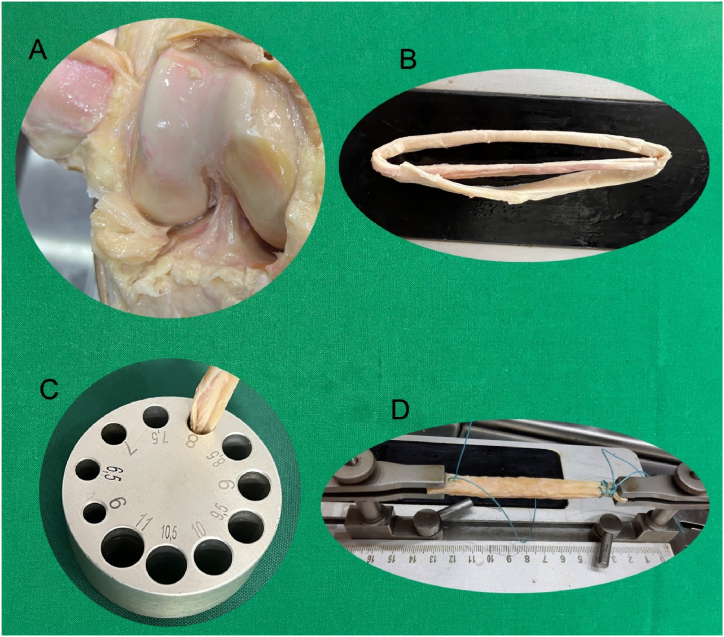
**A:** ACL (Anterior cruciate ligament), **B:** Hamstring tendon (Semitendinosus and gracilis tendons), **C:** Graft size block, **D:** Sample prepared in 8 mm diameter and sutured by ethibond sutures (Graft preparation table).

**Sample size:** A total of 12 cadavers of donors aged ≥18 years (6 male and 6 female) were included in the study. The cadavers were dissected and their ACLs were removed (Fig. 2, Fig. 3A), along with alternative graft options for use in ACL reconstruction procedures, including the Achilles’ tendon (Fig. 2B), patellar tendon (Fig. 2C), hamstring tendon (semitendinosus and gracilis tendons) (Fig. 2, Fig. 3B) and quadriceps tendon (Fig. 2E). The dissected ACL and autograft materials were fixed in 10 % formaldehyde for 3 weeks to reduce tissue fragility. Looney et al. have suggested that grafts with a diameter of at least 8 mm are preferred for tibial screw fixation in anterior cruciate ligament reconstruction (ACLR) due to their lower rates of revision and repeat surgery. As a result, obtaining autografts with a diameter of 8 mm or larger is considered to be a critical surgical requirement [18]. In accordance with the best practices for ACL reconstruction, the grafts obtained were prepared to be 8 mm in size, using a template specifically designed for ACL reconstruction (Graft size block, Tulpar Medical Equipment) (Fig. 3C). To prepare them for ACL reconstruction, the grafts were sutured at both ends using Ethibond sutures (ETHICON) (Fig. 3D).

## 3 Statistical analysis

In the descriptive statistics, continuous variables were expressed as mean and standard deviation, and categorical variables as percentages (range). The normality of the variables was tested for Kurtosis and Skewness, as proposed by Tabachnick and Fidell [19]. Parametric variables were compared with a one-way analysis of variance (ANOVA). For the post-hoc group comparisons, equal variances were assumed and an LSD (Least significant difference) test was applied. The G*power 3.1.9.4 package program was used to determine the sample size of the study, revealing that 12 cadavers (24 ACL, Achilles’, Hamstring, Patella, Quadriceps) would be sufficient to achieve an effect size of over 80 % [20]. IBM SPSS Statistics (Version 24.0. Armonk, NY: IBM Corp.) was used for the statistical analysis, and a p-value less than 0.05 was considered statistically significant.

## 4 Results

**Fig. 4:**
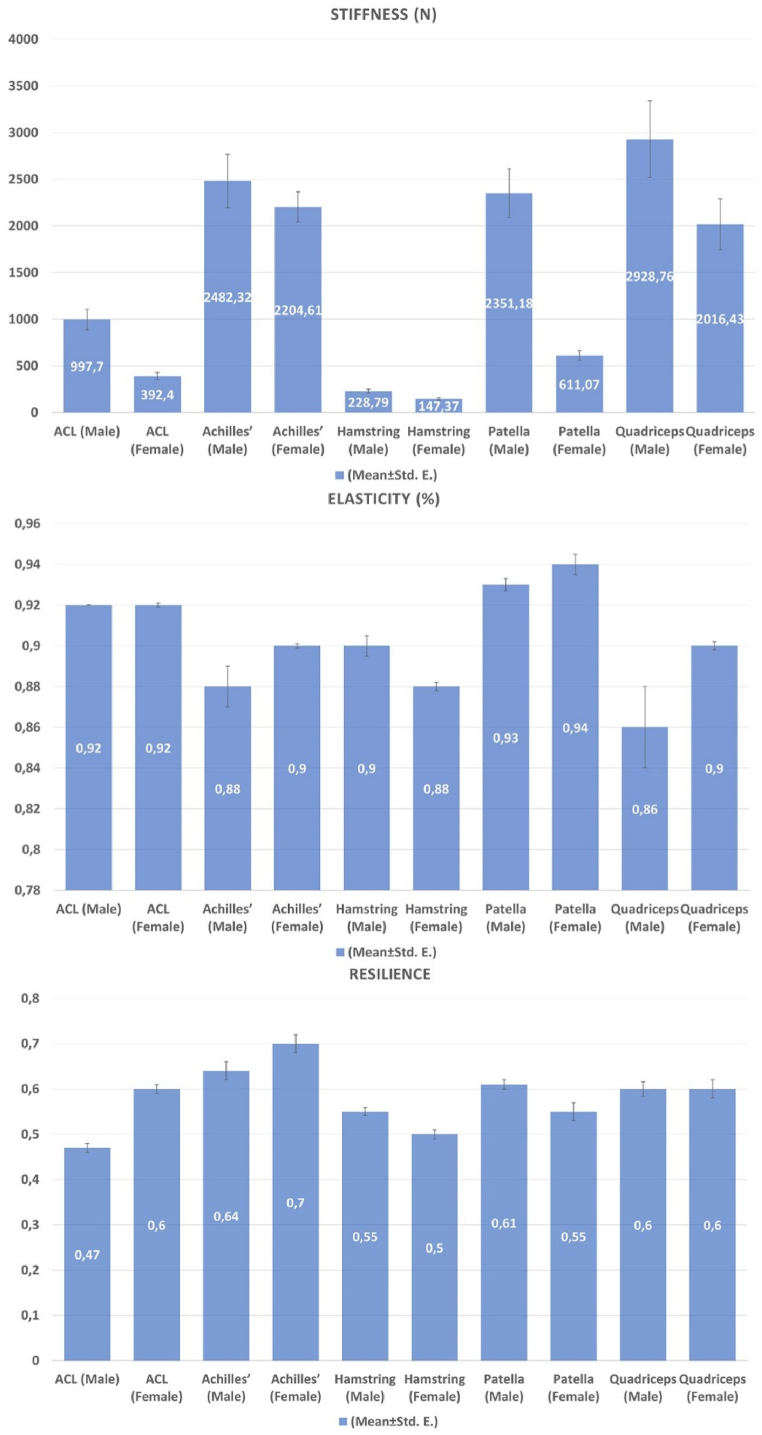
The results of the stiffness, elasticity, resilience analysis of the grafts. (**ACL**: Anterior cruciate ligament).

The results of texture analysis of the ACL and the muscle tendons proposed for use in place of the ACL are presented in Fig. 4.

The stiffness, elasticity, and resilience values of the ACL were first determined, and the graft that most closely resembled the ACL in terms of resilience was the quadriceps tendon graft in females and the hamstring tendon graft in males, suggesting that resilience is among the texture properties (Fig. 4).

As shown in Table 1, since the ANOVA test between tendons for stiffness, elasticity and resilience parameters was significant, LSD was performed as post-hoc analysis. According to the result of this analysis the comparison of the resilience values of the ACL and the grafts recovered from male cadavers, all of the grafts were found to have significantly higher resilience than the ACL (p < 0.001). In females, on the other hand, the Achilles’ tendon graft was found to have significantly higher resilience than the ACL, while the hamstring tendon graft was found to have a significantly lower resilience (p < 0.001 and p = 0.001, respectively). Furthermore, the patellar tendon graft had a lower resilience than the ACL, while the quadriceps tendon graft had similar properties to the ACL, with no statistically significant difference between them (p = 0.163 and p = 0.937, respectively).

**Table 1:** Comparison of ACL and autografts in male and female cadavers in terms of stiffness, elasticity and resilience.

Dependent Variable	Sex	Tendons	Mean ± Std. Error	Min–Max	*P-value*
**Stiffness (N)**	Male	ACL	997,70 ± 109,04	534.65–1741.59	**<0,001**
		Achilles	2482,32 ± 285,53	1081,36–4617,86	
		Hamstring	228,79 ± 23,33	96,63–406,17	
		Patella	2351,18 ± 261,40	1078,43–3473,48	
		Quadriceps	2928,76 ± 411,29	1604,56–5943,70	
	Female	ACL	392,40 ± 37,58	232,05–668,21	**<0,001**
		Achilles	2204,61 ± 161,08	1453,56–3125,56	
		Hamstring	147,37 ± 14,20	78,38–227,35	
		Patella	611,07 ± 55,06	278,22–927,77	
		Quadriceps	2016,43 ± 272,25	1028,94–3878,93	
**Elasticity (%)**	Male	ACL	0,9260 ± 0,0027	0,912–0,937	**0,001**
		Achilles	0,8883 ± 0,0141	0,821–0,945	
		Hamstring	0,9060 ± 0,0059	0,866–0,930	
		Patella	0,9345 ± 0,0038	0,910–0,949	
		Quadriceps	0,8620 ± 0,0241	0,740–0,946	
	Female	ACL	0,9232 ± 0,0016	0,912–0,930	**<0,001**
		Achilles	0,9088 ± 0,0019	0,899–0,918	
		Hamstring	0,8828 ± 0,0027	0,869–0,900	
		Patella	0,9436 ± 0,0052	0,914–0,970	
		Quadriceps	0,9001 ± 0,0024	0,889–0,913	
**Resilience**	Male	ACL	0,4700 ± 0,0172	0,372–0,590	**<0,001**
		Achilles	0,6434 ± 0,0297	0,507–0,780	
		Hamstring	0,5591 ± 0,0085	0,520–0,608	
		Patella	0,6147 ± 0,0100	0,552–0,666	
		Quadriceps	0,6092 ± 0,0161	0,511–0,698	
	Female	ACL	0,6035 ± 0,0144	0,536–0,681	**<0,001**
		Achilles	0,7075 ± 0,0315	0,561–0,850	
		Hamstring	0,5030 ± 0,0112	0,436–0,561	
		Patella	0,5574 ± 0,0232	0,422–0,658	
		Quadriceps	0,6068 ± 0,0279	0,495–0,771	

ACL springiness (Elasticity) is defined as the reversal of a deformation that occurs when a force applied to the ACL is removed. The graft with the elasticity value that most closely resembled that of the ACL, namely the graft with the highest elasticity, was the patellar graft in males (Fig. 4). Only the patellar tendon graft was found to have higher elasticity than the ACL, although the difference was not statistically significant (p = 0.642), while the Achilles’, hamstring and quadriceps tendon grafts were found to be less elastic than the ACL (Fig. 4). The differences between the ACL and hamstring tendon grafts were (p = 0.282) statistically insignificant, while the differences between the Achilles’ and quadriceps tendon grafts were found to be statistically significant (p = 0.045; p = 0.001, respectively). The patellar tendon graft was found to be more elastic than the ACL (p < 0.001) in females, while the Achilles’ (p = 0.002), hamstring (p < 0.001) and quadriceps (p < 0.001) tendon grafts were found to be less elastic than the ACL, and the difference was statistically significant for all grafts.

The stiffness of the Achilles’ tendon graft (392.40 ± 37.58 g) in females and the quadriceps tendon graft in males were found to be higher than that of the ACL. The mean stiffness value of the ACL was 997.70 ± 109.04 g in males, while the Achilles’, quadriceps and patellar tendon grafts were found to be harder, requiring more force to achieve deformation, while the hamstring graft required less force (228.79 ± 23.33 g). The differences in stiffness between the ACL and Achilles’, patellar, quadriceps and hamstring tendon grafts were statistically significant (Table 1) (p < 0.05). Similar to the male samples, the stiffness values of the Achilles’, quadriceps and patellar tendon grafts were higher than that of ACL in the female samples, and the stiffness of the hamstring tendon graft was lower than that of the ACL (Fig. 4). The differences in stiffness values of the ACL and the Achilles’ (p < 0.001) and quadriceps tendon grafts (p < 0.001) were found to be statistically significant, while the differences in the stiffness values of the ACL and the hamstring (p = 0.236) and patellar (p = 0.290) tendon grafts were not statistically significant.

## 5 Discussion

The aim of ACL reconstruction is to reestablish stability in the knee joint so as to prevent further risk of injury to the joint cartilage and meniscus [21]. The patellar and hamstring tendons are the most frequently preferred in ACL reconstruction surgeries [4]. Grafts that are unsuitable for ACL reconstruction may limit the long-term success of surgery, and so using grafts that mimic the natural properties of the ACL is of vital importance for successful outcomes [22]. While grafts used in ACL reconstruction may not completely match the properties of the ACL, they should share the structural and mechanical properties of natural ACLs with minimal antigenicity [23]. It should be noted that optimal graft selection depends not only on the graft properties, but also on the patient's expectations, specifications and activity [23].

Stiffness is defined as the ability to resist any effect applied to the tissue [11] the greater the stiffness of the tissue, the more force is required to produce deformation. Stiffness is thus among the factors affecting tissue endurance, but is also considered to lengthen the average life of the tissue [13]. The stiffness of tissue depends on the material contained within the tissue. In this regard, the stiffness of muscle tissue is determined by the muscle proteins and collagen molecules contained within it. Collagen molecules provide structural support and strength to muscle tissue [24]. Collagen fibrils are relatively elastic and have high resistance against any force, stretching or lengthening, but have the capacity to bend and twist [25,26]. Previous studies identified collagen in different quantities in the anterior cruciate ligament, posterior cruciate ligament, patellar tendon, semitendinosus tendon and gracilis tendons when compared to the amount of collagen found in the tendons in the human knee and its surroundings. Furthermore, the lowest amount of collagen has been identified in the anterior cruciate ligament, while a higher amount of collagen is found in the posterior cruciate ligament, patellar tendon, gracilis tendon and semitendinosus tendon, in ascending order [25]. This study suggests that the quadriceps and Achilles tendons having the highest stiffness values and the ACL having the lowest stiffness values may be related to the amount of collagen in the tissue, as stated in the literature [[24], [25], [26]]. An in vitro study of ACLs revealed that estrogen decreased the concentration of collagen, which may affect the mechanical response of the ACL in different sexes [27]. Similarly, in the present study the stiffness value was higher in males than in females in all tendon grafts, which was attributed by Liu et al. to the lower collagen levels in females than in males due to the presence of estrogen in females [27].

Elasticity refers to the reversal of a deformation resulting from any effect after the effect has been removed [28], while elasticity prevents permanent deformations caused by mechanical factors from affecting tissues. In other words, the more elastic the tissue, the greater the endurance [13]. The ACL should have a more elastic structure if it is to perform its desired functions and to respond to dynamic and mechanical effects [25]. In the present study, the most elastic tendon was found to be the patellar tendon, which had a higher elasticity than both the ACL and the other tendons tested. There was a significant difference between the patellar tendon and the ACL only in women, and it has been suggested in other studies that patellar tendon grafts offer greater stability and lower rates of failure [[29], [30], [31], [32]]. The data obtained in the present study support the finding of Fujii et al. [25] that patellar tendons are more elastic, and that elasticity is a desired factor in grafts to be used in place of ACL, and successful long-term outcomes through the use of these grafts have been reported by Romanini et al. [29], Li et al. [30], Persson and Fevang [31] and Rahr-Wagner et al. [32].

Resilience is defined as the rate of escape of the sample from both the speed and deformation resulting from different forces, and is the parameter defining the ability of the sample to revert to its original form [17]. The resilience of the tissue was calculated as the ratio of the area formed after the maximum peak force during the first compression cycle to the positive force area under the first compression (Fig. 1) [17]. The present study is the first in literature to evaluate tissue resilience and present it as a numerical value that can serve as a marker of tissue recovery status following sudden and powerful mechanical stress. We suggest that this rate should be high in the ACL when making sudden movements and in athletes engaged in sports requiring sudden movement, with resilience values of 0.47 and 0.60 recorded for males and females. The Achilles’ tendon graft offers the highest resilience in both males and females among all grafts with potential for use in place of the ACL, although the selection of the optimal graft should be based not only on the properties of the graft, but also patient expectations, specifications and activities, with the intention being to identify the graft that best resembles the ACL [30]. In this regard, the resilience value may be considered a key parameter as regards the functioning of the ACL in the stabilization of the knee.

In this study, fixed cadavers preserved in 10 % formaldehyde solution were used instead of fresh frozen cadavers. Although it has been reported in some literatures [33] that tissues fixed with formaldehyde partially lose their color and normal tissue properties and that these tissues do not fully reflect normal tissue properties in surgical procedures, formalin-fixed specimens were preferred to reduce tissue fragility and to prevent loss of elasticity and water during thawing. If fresh frozen cadavers had been used in the study, much more realistic results could have been obtained. However, fresh frozen cadavers were not used in this study due to some deformation of the tissue during freezing and defrosting [33].

### 5.1 Limitations of the study

The preparation of grafts to be used in place of the ACL of similar thickness to the ACL limited the evaluation of certain parameters in the TPA. More samples are needed to evaluate whether those with high stiffness values experienced deformation at the points of contact/fixture (endurance), which could not be assessed in the present study due to the limited number of cadavers used. Another limitation is that the specimens used in the study were formalin fixed. Even if formaldehyde-fixed tissues are defective in accurately reflecting surgical procedures, no other method for testing sensitive autografts has been identified.

## 6 Conclusion

In conclusion, this is the first study in which the textural properties of ACL are defined. Among the autografts that may be used in place of the ACL, are, in descending order of endurance, the quadriceps, Achilles, patella and hamstring tendons in males, and the Achilles, quadriceps, patella and hamstring tendons in females. The patella tendon graft was found to have the highest elasticity coefficient compared to ACL in both genders, and the Achilles tendon graft was found to have the highest value of recovery of the original form (Resilience) compared to ACL in both genders. The results of this study suggest that patellar tendon and Achilles tendon grafts may be preferred for ACL reconstruction in both genders according to the parameters evaluated. It is worthy of note that the hamstring tendon, which is mostly preferred for ACL reconstruction procedures, was found to have the lowest value in all parameters. In addition, it is recommended to consider the patient's daily life and expectations when choosing the most appropriate graft. Further studies are needed to examine the textural characteristics of potential autografts, including factors such as cohesiveness, in order to identify ideal options for ACL reconstruction.

## Funding support

This research did not benefit from any grant or funding from agencies in the public, commercial, or not-for-profit sectors.

## Ethical approval

The study was granted approval by the Ethics Committee of Niğde Ömer Halisdemir University (Protocol number: 2021/95).

## CRediT authorship contribution statement

**Ahmet Mert:** Writing – review & editing, Writing – original draft, Methodology, Investigation. **Selim Çınaroğlu:** Writing – review & editing, Writing – original draft, Project administration, Methodology, Investigation. **Murat Aydın:** Writing – review & editing, Writing – original draft, Visualization, Methodology, Investigation. **Fatih Çiçek:** Writing – review & editing, Writing – original draft, Methodology, Investigation, Formal analysis. **Faruk Gazi Ceranoğlu:** Writing – review & editing, Writing – original draft, Methodology, Investigation.

## Declaration of competing interest

The authors declare that they have no known competing financial interests or personal relationships that could have appeared to influence the work reported in this paper.
